# Effects of chemical fertilization on bacterial community in rhizosphere soil of sugarcane

**DOI:** 10.1371/journal.pone.0327545

**Published:** 2025-07-11

**Authors:** Qian Wang, Shang-Tao Jiang, Juan Song, Yi-Hao Kang, Jin-Lian Zhang, Ting-Su Chen, Yang-Rui Li

**Affiliations:** 1 Microbiology Research Institute, Guangxi Academy of Agricultural Sciences, Nanning, China; 2 Jiangsu Key Laboratory for Bioresources of Saline Soils, Jiangsu Synthetic Innovation Center for Coastal Bio-agriculture, School of Wetlands, Yancheng Teachers University, Yancheng, China; 3 Key Laboratory of Sugarcane Biotechnology and Genetic Improvement (Guangxi), Ministry of Agriculture and Rural Affairs/ Guangxi Key Laboratory of Sugarcane Genetic Improvement/ Sugarcane Research Institute, Guangxi Academy of Agricultural Sciences, Nanning, China; Sultan Qaboos University College of Science, OMAN

## Abstract

Most sugarcane growing areas in China have undergone over 30 years of continuous monocropping, and long-term chemical fertilizer application has led to severe soil degradation. In order to provide a theoretical basis for the sustainable development of sugarcane production and scientific and reasonable fertilization, the effects of different fertilization levels on agronomic traits and rhizosphere soil bacterial community in plant crop of sugarcane were investigated. Four fertilization levels were set in the experiment conducted in the field of Guangxi Academy of Agricultural Sciences: no fertilization (0%); low fertilization level (25%); half-reduced fertilization level (50%); and full fertilization level (100%). Two sugarcane varieties GT29 and GT58 were used, and agronomic traits in sugarcane and soil physicochemical properties in the rhizosphere were measured. Illumina high-throughput sequencing technology was employed to analyze the bacterial community structure in soil. The results indicated that the cane yield showed the order of half-reduced fertilization level (50%) > full fertilization level (100%) > low fertilization level (25%) > no fertilization (0%) in both varieties, and that in GT58 showed significant differences in different treatments except for that between half-reduced fertilization level (50%) and full fertilization level (100%). The dominant bacterial phyla in the rhizosphere soil included Proteobacteria, Actinobacteria, Chloroflexi, and Acidobacteria (> 10%). Variance partitioning analysis (VPA) results indicated that soil physicochemical properties had the most significant impact on the bacterial community, followed by chemical fertilization, and then sugarcane variety. Notably, NO_3_^-^, pH, organic matter (OM), available P, Ca, Mg, Fe, Cu and Zn contents in soil all had a significant impact on the bacterial community composition (*P* < 0.05). Differential species analysis revealed that, under high chemical fertilization, three bacterial genera, *Chujaibacter*, *Sporolactobacillus*, and *Ammoniphilus*, were significantly enriched. In contrast, under no fertilization treatment, the bacterial genera such as *Flavisolibacter*, *Aquicella*, *Ramlibacter*, *Anaeromyxobacter*, *Candidatus Solibacter*, *Polycyclovorans*, and *Candidatus Koribacter* were significantly enriched. Co-occurrence network analysis showed that the half-reduced fertilization level (50%) treatment increased both the number of nodes and connections in the co-occurrence network, promoting positive correlations among the bacterial species in the rhizosphere of sugarcane. In summary, under the conditions of this study, compared to the full fertilization treatment, reducing fertilization by half did not decrease cane yield and did not significantly alter the community diversity of rhizosphere soil bacteria. Therefore, it was recommended as a viable alternative for fertilization in sugarcane.

## Introduction

Sugarcane is an important crop for sugar and bioenergy production. It contributes to 80% of the sugar production in the globe, and is cultivated in more than 110 countries [1,2]. China is the third-largest sugar-producing country in the world following Brazil and India [3]. In China, about 90% of sugarcane is planted in southern and southwest areas, mainly in Guangxi and Yunnan provinces [4]. In particular, Guangxi Province is the top sugarcane and sugar producer in China, accounting for more than 65% of the sugar production in the country since 1993 [5]. However, low sugarcane yield is still a major problem in China [6]. To improve sugarcane productivity, chemical fertilizers were overused by the farmers. The annual application of nitrogen in China is as high as 600–800 kg ha^-1^, 8–10 times higher than that in Brazil. Previous studies have confirmed that excessive application of nitrogen fertilizer is not conducive to improving cane yield but reducing the fertilizer use efficiency and the quality of cane in sugarcane [7,8]. At the same time, long-term excessive application of nitrogen fertilizer not only wastes the agricultural resource, but also causes nutrient loss and poses a serious threat to the agricultural ecological environment [9–11]. Therefore, minimal chemical fertilizer inputs for maintaining healthy soil and high crop productivity are urgently needed for commercial sugarcane production in China.

Soil microorganisms are active in terrestrial ecosystems and are one of the key factors affecting the ecological functions of soil organic matter decomposition, nutrient cycling and pest control [12]. They play an important role in maintaining the stability of soil ecosystems and improving soil fertility [13,14]. Soil bacteria are the most abundant soil microorganisms. As the core link in the ecosystem carbon, nitrogen and phosphorus cycle, soil bacteria mineralize nutrient elements through decomposition, form a special inorganic-organic biological complex and release the elements into the soil, promoting the material cycle and energy flow of the ecosystem [15]. Crop rhizosphere soil is the most strongly affected area of “crop-bacteria-soil”; this phenomenon is called rhizosphere soil effect. Rhizosphere soil bacterial community structure and diversity were affected by crop trash returning measures, soil types and crop varieties. Studying the characteristics of rhizosphere soil bacterial community is of great significance for exploring the response of different chemical fertilization models to the agronomic traits of sugarcane and the bacterial community in rhizosphere.

Previous studies have shown that reduction of fertilization in sugarcane had no significant effect on cane quality, sucrose content and soil nutrient content [16]. In recent years, the research on reduction of chemical fertilizer application has mainly focused on soil physicochemical properties, nitrogen balance, crop yield and soil microbial community [17]. However, the effects of reduced fertilization on soil bacterial community structure are still unknown.

Therefore, in this study, a field experiment was conducted to evaluate the impacts of different amounts of fertilizers on the bacterial diversity and community structure in sugarcane rhizosphere soil. The main objectives were to (a) analyze the response of the soil bacterial community structure to different fertilization levels, and (b) evaluate the effect of fertilization amount on the soil physicochemical characteristics and crop yields. The fertilization impact on sugarcane productivity may be closely related with the regulation of the structures and functions of the bacterial community in the rhizosphere, so the results of this research may provide a reference for reasonable fertilization management in sugarcane production.

## Materials and methods

### Sugarcane varieties and experimental design

The seedcanes of sugarcane were planted on January 8, 2020. The experimental field was located in the Lijian Scientific Research Station of Guangxi Academy of Agricultural Sciences in Wuming District, Nanning City, Guangxi (23^o^14’N, 108^o^03’E), covering an area of about 0.67 ha. The soil for experiment is red loam, and the basic physicochemical properties of the soil were as follows: pH 5.1, organic matter 32.3 g kg^-1^, hydrolyzable nitrogen 51.97 mg kg^-1^, available phosphorus 73.2 mg kg^-1^, and available potassium 110 mg kg^-1^.

Two sugarcane varieties were chosen: Gui-Tang 58 (GT58), known for its extensive cultivation, and Gui-Tang 29 (GT29), characterized by strong ratoon and tillering ability. They were provided by Sugarcane Research Institute of Guangxi Academy of Agricultural Sciences. The characteristics of the sugarcane varieties used in this study were as follows [18].

GT58: This variety was derived from the cross of YT85-177 × CP81–1254. It is currently a widely cultivated variety. Its distinctive characteristics include medium plant height, large stalk, rich millable canes, slightly purplish-red leaf sheaths, underdeveloped hair groups, and easy leaf shedding. It has strong ratoon ability, strong adaptability, upright stalk, and lodging resistance, and lacks aerial roots. GT58 is known for high and stable cane yields, intermediate maturity, high sugar content, high resistance to smut disease, but average drought resistance.

GT29: The parentage of this variety is YC94−46 × ROC22. It has strong ratoon ability and can have more than 9 ratoons in production. Its characteristics include erect and compact plant structure, easily detached leaves, intermediate stalk, uniform canes, early ripening, and high sugar content. It has excellent sprouting ability and strong tillering ability, strong cold tolerance and is highly resistant to smut.

The experimental plots were arranged using a split-plot design with four different chemical fertilization levels as subplots, two sugarcane varieties as main plots, and three replicates. This resulted in a total of 48 experimental subplots. Each subplot consisted of five rows, which were 7 m long and 1.2 m between rows. The total area of each subplot was 42 m^2^ (S1 Fig). Due to the differences in tillering of the sugarcane varieties, especially the strong tillering ability of GT29, the planting density was 120,000 buds ha^-1^ for GT58 while 75,000 buds ha^-1^ for GT29. Two rows of the same sugarcane variety were planted between different subplots to serve as isolation zones.

Four different chemical fertilization treatments were designed based on a weighted average of the local farmers’ fertilization practices over the previous three years: T1, no fertilization (0%); T2, low fertilization (25%, 562.5 kg ha^-1^); T3, half fertilization (50%, 1125 kg ha^-1^); and T4, full fertilization (100%, 2250 kg ha^-1^). A controlled-release compound fertilizer with a ratio of N: P: K = 17: 7: 17 was used for fertilization (product of Hebei Tianren Chemical Co., Ltd.). The fertilizers were applied at two stages: 40% was applied as a base fertilizer at planting while the remaining 60% was dressed at the booming growth stage of sugarcane (Supplementary Table S1). The crop was managed the same as for common commercial production.

### Soil sample collection and soil physicochemical property determination

The collection of rhizosphere soil samples was done using the five-point sampling method at harvest on December 5, 2020. The specific procedure for collecting rhizosphere soil was as follows: Large soil clumps were removed before the sampled sugarcane roots were put into sealed plastic bags for transportation on ice to the laboratory. The roots were gently shaken to remove the loose surface soil. A sterile brush was used to collect the soil that adhered closely to the roots, which was considered as rhizosphere soil [19].

The collected rhizosphere soil samples were screened through a 20-mesh sieve, then divided into portions of 800−1000 mg each (in triplicate) and stored at −80°C for soil total DNA extraction. The remaining soil was air-dried naturally, then screened through a 20-mesh sieve, and 300 g of the soil was taken for determination of soil physicochemical properties.

The soil pH was measured using a water-to-soil ratio of 2.5:1. Deionized water was added to the soil, followed by stirring and standing. The pH was measured using a calibrated pH meter. Soil available nitrogen (AN) including ammonium and nitrate nitrogen (NH_4_^+^-N and NO_3_^-^-N) was extracted using a 2 mol L^-1^ potassium chloride (KCl) solution [20] and measured using FOSS Kjeltec™2300 automatic nitrogen analyzer (Danmark). Soil available phosphorus (AP) was extracted using a solution of 0.03 mol L^-1^ NH_4_F and 0.025 mol L^-1^ HCl, and quantified through colorimetry at a wavelength of 880 nm [20]. Soil organic matter (OM), and nitrogen (N), phosphorus (P), potassium (K), calcium (Ca) and magnesium (Mg) in plants were determined by the methods described by Bao [20].

### Determination of agronomic traits

After emergence, the germination rate of sugarcane was investigated on April 10, 2020.

On May 26, 2020, the number of tillers per plant was surveyed. On November 20, 2020, the millable stalks, that is, the plant height was 1.3 m or more, were investigated. On December 5, 2020, the crop was harvested, and 20 plants with similar growth were selected from the middle three rows for trait measurement. Plant height was measured using a tape measure, stalk diameter was measured using a vernier caliper, and hardness was measured using a MYT-1 hardness tester. Brix was measured with PAL-1 portable digital refractometer. Sucrose content was measured with polarimetry following the standard procedure of International Commission for Uniform Methods of Sugar Analysis (ICUMSA) that is commonly applied by sugar mills. Clarified sample solutions were analyzed using a polarimeter (200 mm tube, 20^o^C, sodium light). Results were calculated using the specific rotation of sucrose (+66.5^o^). After harvest, all the canes within the same plot were collected and weighed, and the data were used for cane yield estimation [21].

### DNA extraction, amplification, and sequencing

About 0.5 g of rhizosphere soil sample was used for total DNA extraction following the instructions of the FastDNA Spin Kit for Soil (MP Biomedicals, USA). The DNA concentration and purity were assessed using a NanoDrop 2000 spectrophotometer (Thermo Fisher Scientific, USA). The samples were diluted with sterile water to a concentration of 1 ng µL^-1^ and stored at −80°C freezer. The polymerase chain reaction (PCR) amplification was done with V3-Vpairs 338F (5-ACTCCTACGGGAGGCAGCAG-3) and 806R (5-GGACTACHVGGGTWTCTAAT-3) by an ABI GeneAmp ® 9700 PCR thermocycler (ABI, CA, U.S.A.). The PCR system included 25 µL 2 × Premix Taq, 1 µL primer-F (10 mM), 1 µL primer-R (10 mM), 3 µL DNA (20 ng µL^-1^), and 20 µL nuclease-free water. The PCR procedure was as follows: 94°C denaturation 5 min, 94°C 30 s, 52°C 30 s, 72°C 30 s for a total of 30 cycles, and extension at 72°C for 10 min. The concentrations of PCR products were compared using the GeneTools analysis software (version 4.03.05.0, SynGene). The PCR products from the same sample were mixed and then recovered using a 2% agarose gel. The purified PCR products were isolated using the AxyPrep DNA Gel Extraction Kit (Axygen Biosciences, Union City, CA, USA). A 2% agarose gel electrophoresis was used to verify the quality of the recovered products. Quantification of the recovered products was performed using the Quantus Fluorometer (Promega, USA). Library preparation was done using NEXTFLEX Rapid DNA-Seq Kit: (1) Adapter ligation: Adapters were ligated to the PCR products. (2) Magnetic bead selection: Removal of adapter dimers was done by magnetic bead selection. (3) Library template enrichment via PCR amplification: Enrichment of library templates was done through PCR amplification. (4) Magnetic bead recovery of PCR products: Final library was obtained through magnetic bead recovery of PCR products. Sequencing was conducted on the Illumina MiSeq PE250 platform by Shanghai Majorbio Bio-pharm Technology Co., Ltd., China.

### Bioinformatics and statistical analyses

The raw high-throughput sequencing data were subjected to quality control using the Fastp software (Version 0.20.0) [22]. Sequence merging was performed using the FLASH software (Version 1.2.7) [23], and clustering of sequences into operational taxonomic units (OTUs) at a 97% similarity level was conducted using the UPARSE software (Version 7.1) [24]. Chimeric sequences were removed during this process. Taxonomic classification of the sequences was carried out using the RDP classifier algorithm (Version 2.2) [25]. The sequences were compared on the Silva 16S rRNA database (v138), resulting in taxonomic information corresponding to each OTU. The α diversity (Chao richness and Shannon’s diversity) statistics was analyzed for each sequencing sample using Usearch alpha_div (version 10.0.240). Statistical analysis of the sequencing data was conducted in the R environment (version 3.5.1). The β diversity (principal coordinate analysis, PCoA) based on Bray Curtis dissimilarities was analyzed using the R package “vegan”. Redundancy analysis (RDA) was conducted using Canoco V5.0 to examine the relationships between the physicochemical properties of sugarcane rhizosphere soil and bacterial community diversity. For each fertilization treatment, the top 100 abundant bacterial species were selected to construct a co-occurrence network. The Spearman’s correlations at R^2^ > 0.6 and *p* < 0.05 were used for the network construction. Functional Annotation of Procaryotic Taxa (FAPROTAX) analysis was applied to predict bacterial functions in soil samples. After obtaining 16S rRNA gene sequences and classifying OTUs, the FAPROTAX script was used to match the taxonomic data of functional pathways [26]. In terms of agronomic traits and soil properties, the significance of differences between the fertilization treatments was evaluated by Duncan’s multiple comparison method. Data processing and visualization were performed using Microsoft Excel 2017, SPSS 20.0, and the R programming language.

## Results and analysis

### Impact of different chemical fertilization levels on the agronomic traits of sugarcane

**Fig 1:**
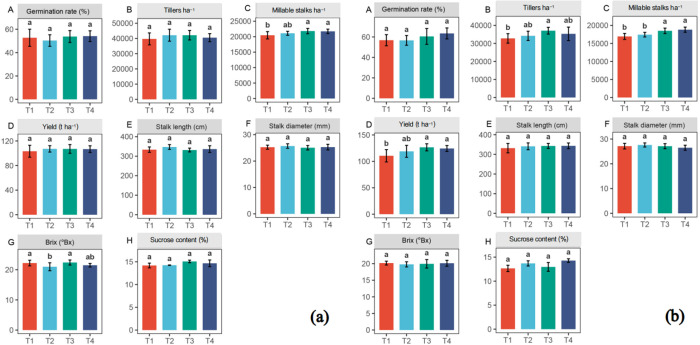
Agronomic characters in sugarcane variety GT29 (a) and GT58 (b). A, germination rate; B, number of tillers; C, number of millable stalks; D, Yield; E, stalk length; F, stalk diameter; G, Brix; H, sucrose content; T1, no fertilization (0%); T2, low fertilization (25%); T3, half fertilization (50%); T4, full fertilization (100%).

The data in Fig 1 showed that chemical fertilization tended to improve the performances of the agronomic traits in both sugarcane varieties GT29 and GT58 although most of them were not statistically different in different treatments, and the effects on GT58 were more significant compared with GT29. For GT29, only millable stalks ha^-1^ showed significant differences between T2, T3, T4, and T1 (Fig 1a). For GT58, there were significant differences in tiller numbers between T2, T3, T4, and T1, in millable stalks ha^-1^ between T3, T4, and T1, T2, and in cane yield between T3, T4, T2, and T1 (Fig 1b).

### Impact of different chemical fertilization levels on soil physicochemical properties in sugarcane rhizosphere soil

The data in Table 1 showed that chemical fertilization tended to improve the the OM, AP and AK levels in the rhizosphere soil of both sugarcane varieties GT29 and GT58. For GT29, significant differences were observed in pH between T1 and T4 Treatments, and in AP between T4 and the other three treatments. For GT58, significant differences were observed in TN between T4 and T1, T2, in AP between T4 and the other three treatments, and in AK between T4 and T1, T2. These results indicate that the different chemical fertilization levels had relatively stronger effects on the physicochemical properties of the rhizosphere soil of GT58 than GT29.

**Table 1:** Effects of different chemical fertilization on the physicochemical properties of sugarcane rhizosphere soil.

Fertilization level	Sugarcane variety	pH	OM (g kg^-1^)	NH_4_^ + ^-N (mg kg^-1^)	NO_3_^—^N (mg kg^-1^)	AP (mg kg^-1^)	AK (mg kg^-1^)
T1	GT29	4.38 ± 0.15a	27.8 ± 6.2a	6.15 ± 1.69a	6.1 ± 5.47a	20.5 ± 6.6b	120.5 ± 50.7a
T2		4.23 ± 0.07ab	28.1 ± 5.1a	7.23 ± 1.51a	4.51 ± 2.22a	39.9 ± 15.9b	126.5 ± 29.7a
T3		4.3 ± 0.25ab	31.0 ± 4.2a	5.94 ± 1.15a	5.04 ± 3.78a	26.8 ± 14.0b	154.9 ± 51.7a
T4		4.15 ± 0.09b	30.1 ± 3.9a	5.74 ± 1.11a	6.25 ± 3.2a	73.3 ± 36.9a	166.1 ± 25.2a
T1	GT58	4.34 ± 0.08a	24.7 ± 5.3a	8.37 ± 7.05a	2.45 ± 1.55b	22.9 ± 9.7b	102.3 ± 16.8b
T2		4.28 ± 0.06a	28.0 ± 6.5a	6.29 ± 3.2a	2.0 ± 2.02b	24.4 ± 5.6b	99.7 ± 32.8b
T3		4.3 ± 0.17a	27.2 ± 5.3a	6.86 ± 1.9a	5.05 ± 3.56ab	30.9 ± 9.7b	136.2 ± 51.9ab
T4		4.2 ± 0.14a	30.4 ± 3.0a	7.23 ± 2.9a	5.74 ± 2.52a	68.1 ± 47.3a	171.7 ± 27.7a

Note: Values are means±standard deviation (n = 6). Different letters mean significant differences (*P* < 0.05). T1, no fertilization (0%); T2, low fertilization (25%); T3, half fertilization (50%); T4, full fertilization (100%).

Analysis of soil micro elements showed that chemical fertilization tended to increase the levels of Fe and Zn but decrease the levels of Mg, Cu, and Ca in the rhizosphere soil of both sugarcane varieties GT29 and GT58 (Table 2). For GT58, Fe demonstrated a significantly higher level in T4 than in the other treatments. For GT29, Fe content was significantly higher in T4, Mn content showed the highest in T2, while Mg content was higher in T1 than in T3 and T4, and Ca content was higher in T1 than in T4 (Table 2).

**Table 2:** Effects of different chemical fertilization on the microelements in sugarcane rhizosphere soil.

Fertilization level	Sugarcane variety	Fe (mg kg^-1^)	Mg (mg kg^-1^)	Cu (mg kg^-1^)	Zn (mg kg^-1^)	Mn (mg kg^-1^)	Ca (mg kg^-1^)
T1	GT29	20.9 ± 4.0b	41.2 ± 2.8a	0.65 ± 0.10a	0.74 ± 0.15a	14.4 ± 3.9b	596.5 ± 79.1a
T2		22.5 ± 4.8b	37.3 ± 5.4ab	0.62 ± 0.08a	0.8 ± 0.12a	21.9 ± 5.6a	483.4 ± 71.0ab
T3		20.9 ± 5.1b	33.8 ± 4.5b	0.55 ± 0.11a	0.69 ± 0.18a	13.6 ± 8.3b	496.6 ± 153.4ab
T4		30.3 ± 7.0a	33.2 ± 7.0b	0.61 ± 0.18a	0.77 ± 0.19a	11.6 ± 3.2b	437.2 ± 96.6b
T1	GT58	21.8 ± 3.4b	39.5 ± 8.1a	0.63 ± 0.08a	0.66 ± 0.06a	18.5 ± 3.1a	535.3 ± 64.1a
T2		22.0 ± 4.7b	34.2 ± 9.0a	0.58 ± 0.07a	0.73 ± 0.14a	21.3 ± 10.7a	467.9 ± 80.7a
T3		21.6 ± 5.7b	37.7 ± 7.5a	0.52 ± 0.13a	0.69 ± 0.12a	13.5 ± 7.3a	481.9 ± 108.0a
T4		29.1 ± 6.0a	37.1 ± 10.9a	0.59 ± 0.06a	0.87 ± 0.32a	13.6 ± 4.4a	444.7 ± 146.2a

Note: Values are means ± SD (n = 6). Different letters mean significant differences (*P* < 0.05). T1, no fertilization (0%); T2, low fertilization (25%); T3, half fertilization (50%); T4, full fertilization (100%).

### Analysis of α diversity of bacteria in sugarcane rhizosphere at different chemical fertilization levels

Good’s coverage indices for all samples were above 98%, indicating that the sequencing data were good enough to reflect the complete diversity of each sample (Table 3). For different chemical fertilization treatments, the differences were small in Shannon, Ace, and Chao1 indices of soil bacteria in the rhizosphere for both sugarcane varieties GT29 and GT58.

**Table 3:** Indices of soil bacterial diversity and richness in sugarcane fields under different chemical fertilization conditions.

Variety	Fertilization	Shannon	Ace	Chao1	Coverage (%)
GT29	T1	6.42 ± 0.06a	2238 ± 133a	2254 ± 115a	98.1a
	T2	6.33 ± 0.18a	2228 ± 123a	2249 ± 147a	98.0a
	T3	6.43 ± 0.1a	2296 ± 99a	2280 ± 109a	99.0a
	T4	6.31 ± 0.12a	2194 ± 150a	2204 ± 145a	98.0a
GT58	T1	6.42 ± 0.09a	2214 ± 125a	2235 ± 137a	98.2a
	T2	6.39 ± 0.1a	2221 ± 102a	2235 ± 89a	98.1a
	T3	6.45 ± 0.15a	2268 ± 119a	2279 ± 116a	98.0a
	T4	6.27 ± 0.15a	2124 ± 180a	2121 ± 190a	98.1a

Note: Data in the table are mean ± SD (n = 6). Values followed by different small letters mean significant difference between different chemical fertilization treatments (*P *< 0.05). T1, no fertilization (0%); T2, low fertilization (25%); T3, half fertilization (50%); T4, full fertilization (100%).

### Composition of bacterial communities in sugarcane rhizosphere soil at different chemical fertilization levels

**Fig 2:**
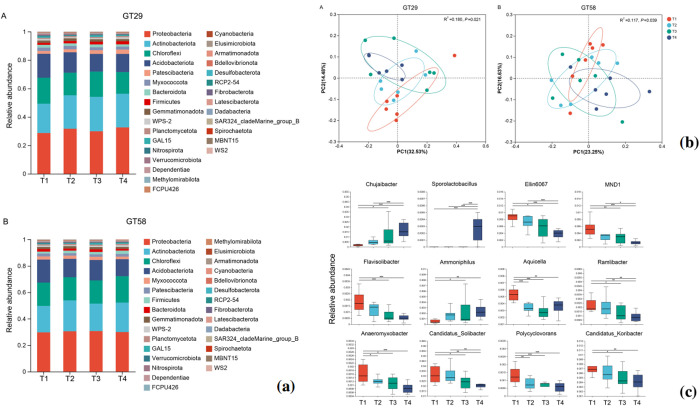
Relative abundance of major bacterial phyla (a), principal coordinates analysis (PCoA) of the relative abundance of bacteria at the OTU level (b), and genera of differential bacteria (c) in rhizosphere of sugarcane on different chemical fertilization levels. T1, no fertilization (0%); T2, low fertilization (25%); T3, half fertilization (50%); T4, full fertilization (100%).

After removing the low-quality sequences, a total of 1,111,632 high-quality bacterial 16S rRNA gene V3-V4 sequences with 23,159 sequences per library on average were obtained from Illumina MiSeq sequencing. 99.8% of the sequences had lengths ranging from 401 to 420 bp. A rarefaction curve was constructed using the random sampling method, plotting the number of sequences sampled against the corresponding number of OTUs (S2A Fig). The rarefaction curve showed that, after a certain sequencing depth, the number of OTUs detected in each sample reached a plateau but did not reach saturation. The sequencing coverage for all the samples was above 99%, indicating that the sequencing data were sufficient to represent the microbial communities in the samples. The Shannon-Wiener curve (S2B Fig) also indicated that the sequencing depth was adequate. The taxonomic composition of soil bacteria at the phylum level in different treatments was shown in Fig 2. The data in Fig 2 showed that the taxonomic composition at phylum level was very similar in different treatments. Proteobacteria, Actinobacteria, Chloroflexi, and Acidobacteria were the most abundant phyla in all the treatments.

For GT29 (Fig 2a), the relative abundances of Proteobacteria, Actinobacteria, Chloroflexi, and Acidobacteria were 30.73% (28.69%−32.63%), 23.01% (20.62%−24.21%), 16.79% (15.20%−18.24%), and 14.14% (12.49%−16.88%), respectively. Compared to the full fertilization treatment (T4), the relative abundances of Chloroflexi and Acidobacteria were significantly increased in the low fertilization (T2) and half fertilization (T3) treatments. The differences in the relative abundances of Proteobacteria and Actinobacteria were not significant between these treatments. However, when compared to the no-fertilization treatment (T1), the relative abundances of Proteobacteria and Actinobacteria were significantly increased in the low fertilization (T2) and half fertilization (T3) treatments.

For GT58 (Fig 2a), the relative abundances of Proteobacteria, Actinobacteria, Chloroflexi, and Acidobacteria were 30.21% (29.71%−30.69%), 21.58% (19.96%−23.36%), 18.2% (17.52%−19.99%), and 14.91% (12.88%−17.37%), respectively. When compared to the control with full fertilization treatment (T4), the relative abundances of Proteobacteria, Actinobacteria, Chloroflexi, and Acidobacteria were significantly increased in the low fertilization (T2) and half fertilization (T3) treatments. Compared to the no fertilization treatment (T1), the relative abundances of Proteobacteria, Actinobacteria, and Chloroflexi were significantly increased in the low fertilization (T2) and half fertilization (T3) treatments, while the relative abundance of Acidobacteria was significantly decreased.

### β diversity (PCoA) at different chemical fertilization levels

Based on the information of OTUs, the results of PCoA revealed that the fertilization level significantly influenced the community composition in the rhizosphere soil of both sugarcane varieties (Fig 2b). It can be observed that T1 and T4 treatments could be clearly distinguished, while there was no clear separation for T1, T2, and T3 treatments. The impact of fertilization levels on the bacterial communities appears to be more pronounced in variety GT29 compared to GT58.

### Differential species analysis

Differential species analysis was conducted using the Kruskal-Wallis H test to examine the differences in bacterial genera among the chemical fertilization treatments. As indicated in Fig 2c, with the increase in chemical fertilization levels, the relative abundance of the genus *Chujaibacter* was significantly increased from 0.23% in T1 to 1.68% in T4. The relative abundance of the genus *Sporolactobacillus* also was significantly increased from 0.0007% in T1 to 0.03% in T4. Similarly, the relative abundance of the genus *Ammoniphilus* was significantly increased from 0.05% in T1 to 0.24% in T4. This implies that, under high chemical fertilization condition, the bacterial genera *Chujaibacter*, *Sporolactobacillus* and *Ammoniphilus* showed more significant enrichment. Conversely, under no chemical fertilization condition, the bacterial genera *Flavisolibacter*, *Aquicella*, *Ramlibacter*, *Anaeromyxobacter*, *Candidatus Solibacter*, *Polycyclovorans*, and *Candidatus Koribacter* exhibited more significant enrichment.

### The relationship between bacterial community and soil physicochemical properties

**Fig 3:**
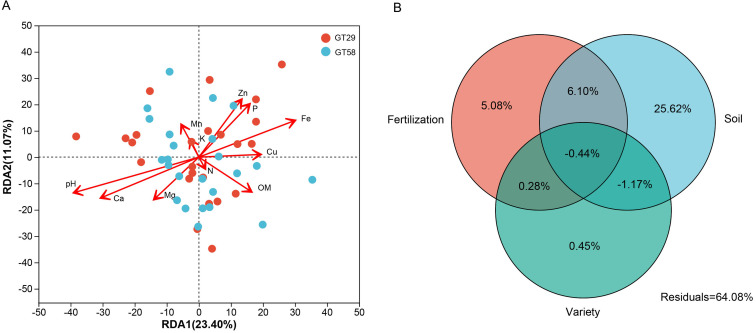
Distance-based RDA (A) and variance decomposition analysis (VPA) (B) results reflecting the influences of different fertilization treatments and environmental factors on the rhizosphere bacterial communities at genus level. The arrows represent environmental factors, and the arrow length represents the degree of influence.

To investigate the influence of different fertilization levels on soil physicochemical properties and the impact on rhizosphere bacterial community structure, RDA was conducted to assess the relationship between the microbial communities at the genus level and soil factors. The data in Fig 3A showed that the diversity of bacterial communities in sugarcane rhizosphere at the genus level under different chemical fertilization conditions explained 23.45% and 12.29% of the variation on two axes, indicating that environmental factors could largely explain the differences in soil bacterial communities. The results of RDA (Fig 3A) and Mantel tests (Supplementary Table S2) revealed that soil variables such as NO_3_^-^, pH, OM, P, Ca, Mg, Fe, Cu, and Zn had significant effects on the bacterial communities. However, there was no significant difference between the two sugarcane varieties. To further explore the impact of overall environmental factors and potential unaccounted factors on the rhizosphere bacterial community structure, VPA was conducted. The VPA results (Fig 3B) showed that the explanatory power of soil factors was 25.62%, indicating that the environmental factors had a strong impact on the rhizosphere bacterial community structure. Specifically, the influence was in the order of soil (25.62%)> fertilization level (5.08%)> sugarcane variety (0.45%).

### Network correlation analysis

**Fig 4:**
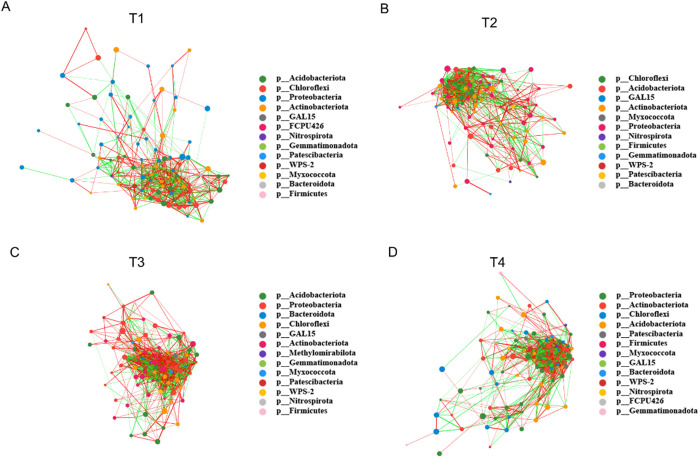
Network analysis of bacteria in sugarcane rhizospheres in different fertilization treatments. T1, no fertilization (0%); T2, low fertilization (25%); T3, half fertilization (50%); T4, full fertilization (100%).

A species correlation network was constructed based on the top 100 abundant bacterial species in different fertilization treatments. Overall, the complexity of the bacterial network varied with the chemical fertilization levels, showing an initial increase followed by a decrease (increase from 0% to 50% fertilization level and decrease from 50% to 100% fertilization level). For the number of edges (connections) in the networks for each fertilization level, the total edges were 675 (371 positive edges, 304 negative edges) in T1 (Fig 4A), 897 (447 positive edges, 450 negative edges) in T2 (Fig 4B), 1190 (643 positive edges, 547 negative edges) in T3 (Fig 4C), and 1036 (538 positive edges, 498 negative edges) in T4 (Fig 4D). Notably, the level T3 exhibited the most nodes and connections, indicating the most positive correlations in the co-occurrence network. This suggests that appropriate fertilization can promote more beneficial interactions between the bacterial species. Analysis of network parameters showed that the network density and clustering coefficient gradually increased with the increasing of chemical fertilization level but then began to decrease after reaching a threshold. In contrast, the average path length and modularity gradually decreased with the increasing of chemical fertilization levels and then began to increase after reaching a threshold (Supplementary Table S3).

### Functional prediction of soil bacterial community using FAPROTAX

**Fig 5:**
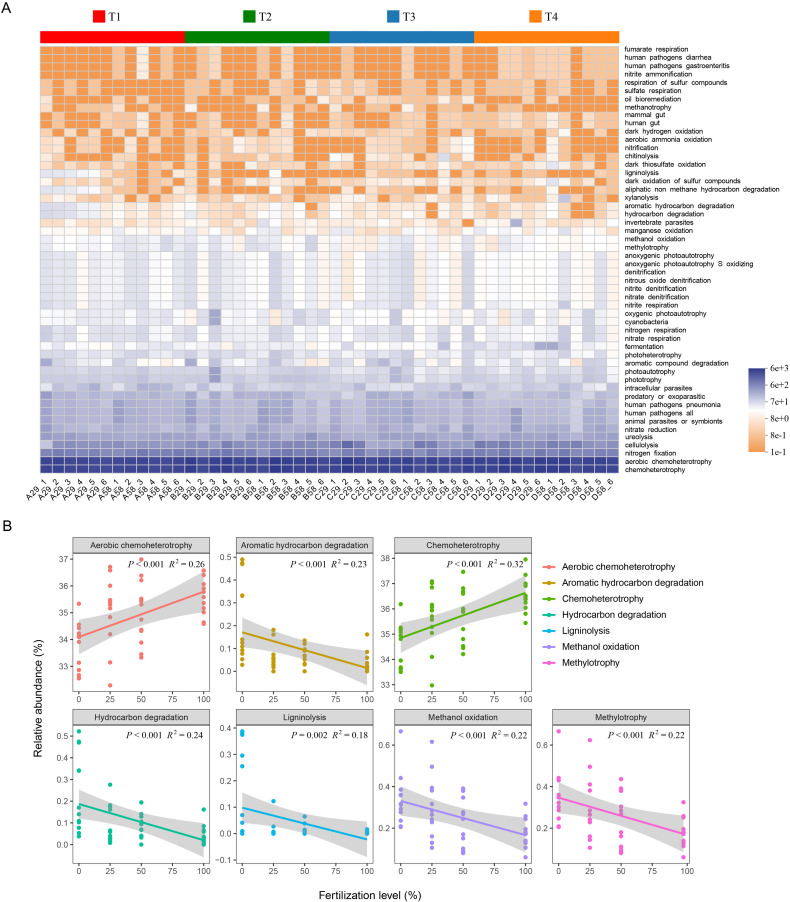
Prediction for the ecological functional composition of the bacterial community from Functional Annotation of Procaryotic Taxa (FAPROTAX) (A) and relationship between fertilization level and related functions (B). Values are the means±SD (n = 6). Data with different letters are significantly different (*P* < 0.05). T1, no fertilization (0%); T2, low fertilization (25%); T3, half fertilization (50%); T4, full fertilization (100%).

Based on the bacterial functional groups, a hierarchical clustering heatmap of bacterial functional group relative abundances was generated to reflect the differences in the functional profiles of the rhizosphere soil bacteria under different chemical fertilization conditions. As shown in Fig 5A, a total of 52 ecological functions were obtained from FAPROTAX functional annotation. For all the treatments, chemoheterotrophy and aerobic chemoheterotrophy showed the highest expression levels. Some metabolic functions such as nitrite respiration, nitrate denitrification, nitrite denitrification, nitrous oxide denitrification, denitrification, anoxygenic photoautotrophy S oxidizing, anoxygenic photoautotrophy and nitrification decreased with the increase of fertilization level, but cellulolysis, nitrate reduction and nitrite ammonification increased with the increase of fertilization level. In terms of correlations between chemical fertilization level and functional categories, there were positive correlations of chemical fertilization level with aerobic chemoheterotrophy and chemoheterotrophy functions, and negative correlations of chemical fertilization level with the functions of aromatic hydrocarbon degradation, hydrocarbon degradation, ligninolysis, methanol oxidation, and methylotrophy (Fig 5B).

## Discussion

### Impact of different chemical fertilization levels on sugarcane agronomic traits

Crop yield is a comprehensive reflection of various agronomic traits and is often a primary focus in agriculture. Increasing crop yield is a main goal in agricultural production. In traditional sugarcane cultivation, many farmers believe that fertilization is crucial for increasing sugarcane productivity. Consequently, they often apply fertilizers excessively, sometimes without considering the cost [24–26]. Despite the gradual improvement in nutrient use efficiency in China over the last decade, a massive amount of inorganic fertilizers such as nitrogen, phosphorous, and potassium have been applied to farmland to improve crop yields, resulting in a slew of serious ecological issues like soil organic matter loss, low soil fertility, nutrient imbalance, and soil degradation [27–29].

Studies have shown that increasing nitrogen fertilizer application can promote sugarcane growth and enhance cane yield, but it is not a case of “the more fertilizer, the better”, rather, yield might decline after reaching a threshold [30–32]. This aligns with the results of this study. In this study, fertilization showed a positive effect on cane yield for sugarcane. Especially for the variety GT58, the T3 and T4 treatments resulted in significant cane yield increases by 12.67% and 11.11%, respectively, compared to the no fertilization (T1) treatment (*P* < 0.05) while no significant differences were found between the T2 and T3 or T4 treatments. This suggests that cane yield did not increase with higher fertilizer application levels after a threshold, instead excessive fertilization might lead to yield decrease. However, the effect of different chemical fertilization treatments on cane yield in GT29 was not as good as in GT58, possibly because GT29 is a variety with lower fertilizer requirements.

### Drivers of soil bacterial community changes

In this study, results of VPA showed that the regulation of soil physicochemical properties on the rhizosphere microbial community structure of sugarcane was much higher than that of fertilization and variety, which confirmed the hierarchical regulation theory of soil-microorganism-plant interaction system [33]. Soil ecosystems are formed by complex interactions between biological communities and physicochemical variables, which jointly determine the overall quality of soil [34]. The complex regulatory network of the soil bacterial community is closely related to soil fertility and ecological function, especially soil pH [35,36]. Previous studies showed that the compositions of bacterial communities were significantly correlated with soil characteristics such as pH, OM, AP, and AK [37–40]. This study confirmed that pH and the contents of OM, AP, Ca, Mg, Fe, Cu, and Zn in soil were significantly correlated with the changes in the bacterial community structure in rhizosphere of sugarcane.

### Impact of different chemical fertilization levels on soil bacteria community

The response mechanism of microbial community to fertilization and its ecological function evolution are important research directions of soil microbial ecology. In this study, PCoA analysis showed that fertilization significantly changed the microbial community composition (*P* < 0.05), which was highly consistent with the effect of nitrogen addition on soil microbial β diversity [41]. Specifically, the enrichment of Proteobacteria and Actinobacteria in the high fertilization treatment reflected their adaptability to high chemical fertilization environments. As a typical copiotrophs, Proteobacteria is rich in nitrogen metabolism-related genes (such as *amoA* and *nirK*) in its genome, which can quickly utilize soluble nutrients [42]; Actinobacteria has a significant competitive advantage under fertilization conditions due to its unique ability of antibiotic synthesis and complex organic matter degradation [30]. On the contrary, the decrease of Acidobacteria and Chloroflexi in the high fertilization treatment implied the competitive disadvantage of oligotrophs under the high chemical fertilization environment, which was consistent with the decreasing trend of Acidobacteria abundance observed in long-term fertilization experiments in tropical soils [43].

Previous research reported that root irrigation with Sporolactobacillus inoculants can enhance substrate enzyme activity, accelerate the supply of rhizosphere available nutrients, increase the content of rhizosphere available nitrogen, and promote nutrient absorption, translocation, assimilation, and accumulation in plants, ultimately improving nutrient utilization efficiency [44]. The differential species analysis in this study revealed that, under high chemical fertilization condition, the bacteria genera *Chujaibacter*, *Sporolactobacillus*, and *Ammoniphilus* were significantly enriched (Fig 2C). This aligned with the global patterns of copiotroph proliferation under nutrient-rich conditions, that is, *Chujaibacter* can promote plant growth and nutrient cycling [45]. *Sporolactobacillus* forms stress-resistant endospores that tolerate osmotic shock from high-salinity fertilizers and secrets ACC deaminase to alleviate ethylene-induced plant stress [46]. *Ammoniphilus* thrives via ammonification in N-enriched environments [47]. This study found that under low chemical fertilization condition, the bacteria genera *Flavisolibacter*, *Aquicella*, *Ramlibacter*, *Anaeromyxobacter*, *Candidatus Solibacter*, *Polycyclovoran*s, and *Candidatus Koribacter* were significantly enriched, and the bacterial interactions were enhance. This aligned with the ecological theory on resource-mediated networking. The moderate nutrient supply treatment (T3) promoted facilitative niche partitioning, where genera like *Chujaibacter* and *Candidatus Solibacter* (C-degraders) form synergistic relationships [48]. Conversely, the excessive fertilization treatment (T4) intensified competitive exclusion, reducing the network stability.

This study is complementary to the report by Niu et al. [21] on the core conclusions. The study of Niu et al. [21] was conducted in a sugarcane field with strong acidity (pH 4.18) with two different levels of organic water soluble fertilizer containing fulvic acid, amino acid and other components, and the results found the organic water soluble fertilizer treatments alleviated soil acidity, increased the organic matter, dissolved organic carbon content, and available potassium contents in the soil, and further improved agronomic parameters and sugarcane yield compared with conventional inorganic fertilization. Both treatments significantly increased the species richness of microorganisms, especially the enrichment of beneficial symbiotic bacteria such as Acidobacteria and Planctomycetes, which are more conducive to the healthy growth of plants. In the present study, the experiment was conducted on moderately acidic soil (pH 5.1) with different levels of inorganic NPK compound fertilizer. This study found the abundance of bacterial genus *Chujaibacter* (with growth-promoting potential) (Fig 2C), the co-occurrence network complexity of bacterial flora (Fig 4C) were increased significantly under half-amount chemical fertilizer (T3) treatment. Based on the results of the present study, the half-amount of chemical fertilizer treatment (T3) maintained the cane yield by avoiding nutrient imbalance (such as Fe/Zn accumulation), selectively enriching functional bacteria (such as *Chujaibacter*), and optimizing the microbial interaction network, while full-amount chemical fertilizer treatment reduced the network complexity of bacterial flora. In summary, both strategies of organic water soluble fertilizer application and chemical fertilizer reduction are good for improving soil ecology but their effects on soil microbial communities are quite different.

In this study, through FAPROTAX, a total of 52 ecological functions were found, and the highest expression levels were observed for chemoheterotrophy and aerobic chemoheterotrophy. Some metabolic functions such as nitrite respiration, nitrate denitrification, nitrite denitrification, nitrous oxide denitrification, denitrification, anoxygenic photoautotrophy S oxidizing, anoxygenic photoautotrophy and nitrification decreased with the increase of fertilization level, but cellulolysis, nitrate reduction and nitrite ammonification increased with the increase of fertilization level. It should be pointed out that FAPROTAX prediction has limitations such as high functional redundancy and rough pathway analysis [49]. In the future, it is necessary to combine metagenomic binning technology to analyze the functional contribution of key strains such as *Sporolactobacillus* nitrogen transformation pathway.

## Conclusion

The results in this study demonstrated that half fertilization (T3, 1125 kg ha^-1^ controlled-release compound fertilizer with a ratio of N: P: K = 17: 7: 17) is good enough to achieve healthy growth and high yield in sugarcane, indicating the feasibility of rational fertilization reduction. The rhizobacterial communities were dominated by Proteobacteria, Actinobacteria, Chloroflexi, and Acidobacteria at phylum level (>10%), with their structural composition primarily driven by soil physicochemical properties (NO_3_^-^, pH, organic matter, etc.), followed by fertilization levels, and minimally influenced by sugarcane cultivars. In T3 treatment, beneficial genera such as *Chujaibacter* were significantly enriched, accompanied by enhanced co-occurrence network complexity and bacterial interactions. A total of 52 ecological functions were found through FAPROTAX. In conclusion, under the conditions of the present study, half fertilization (T3, 1125 kg ha^-1^ controlled-release compound fertilizer with a ratio of N: P: K = 17: 7: 17) not only sustained sugarcane productivity but also optimized the rhizobacterial interactions and functional equilibrium to achieve soil microecological homeostasis, providing a theoretical foundation for green fertilizer-saving management in sugarcane production.

## Supporting information

Supplementary Table S1 The fertilization treatments and their corresponding amounts applied in sugarcane. (DOCX)

Supplementary Table S2 Mantel test. (DOCX)

Supplementary Table S3 Network analysis of related parameters. (DOCX)

Fig S1 Field experiment design of different fertilization levels. (DOCX)

Fig S2 Dilution curves. (DOCX)

## Abbreviations

PCR: polymerase chain reaction
FAPROTAX: Functional Annotation of Procaryotic Taxa
PCoA: principal coordinates analysis
VPA: Variance partitioning analysis
