# Supplementary material for: Effects of chemical fertilization on bacterial community in rhizosphere soil of sugarcane
Source: PLoS One. 2025 Jul 11;20(7):e0327545. doi: 10.1371/journal.pone.0327545 (PMC12250518; doi:10.1371/journal.pone.0327545)
Supplement: Supplementary Table S1 — (DOCX) [file pone.0327545.s001.docx]

**Supplementary Table** **S1.** **The fertilization treatments and their corresponding amounts applied in sugarcane.**

| Treatment | Fertilization level | Total fertilizer  (kg ha^-1^) | Base fertilizer  (kg ha^-1^) | Dressed fertilizer  (kg ha^-1^) |
| --- | --- | --- | --- | --- |
| T1 | No fertilization (0%) | 0 | 0 | 0 |
| T2 | Low fertilization (25%) | 562.5 | 225 | 337.5 |
| T3 | Half fertilization (50%) | 1125 | 450 | 675 |
| T4 | Full fertilization (100%) | 2250 | 900 | 1350 |
