# Supplementary material for: Effects of chemical fertilization on bacterial community in rhizosphere soil of sugarcane
Source: PLoS One. 2025 Jul 11;20(7):e0327545. doi: 10.1371/journal.pone.0327545 (PMC12250518; doi:10.1371/journal.pone.0327545)
Supplement: Supplementary Table S2 — (DOCX) [file pone.0327545.s002.docx]

**Supplementary Table S2. Mantel test.**

| Soil factor | R^2^ | *p* value |
| --- | --- | --- |
| N-NH_4_^+^ | 0.011 | 0.774 |
| N-NO_3_^-^ | 0.126 | 0.045 |
| P | 0.264 | 0.002 |
| K | 0.013 | 0.725 |
| Ca | 0.540 | 0.001 |
| Mg | 0.183 | 0.012 |
| Fe | 0.504 | 0.001 |
| Mn | 0.053 | 0.297 |
| Cu | 0.166 | 0.015 |
| Zn | 0.252 | 0.003 |
| OM | 0.157 | 0.019 |
| pH | 0.799 | 0.001 |
