# Supplementary material for: Effects of chemical fertilization on bacterial community in rhizosphere soil of sugarcane
Source: PLoS One. 2025 Jul 11;20(7):e0327545. doi: 10.1371/journal.pone.0327545 (PMC12250518; doi:10.1371/journal.pone.0327545)
Supplement: Supplementary Table S3 — (DOCX) [file pone.0327545.s003.docx]

**Supplementary Table S3. Network analysis of related parameters.**

| Network topological characteristic | T1 | T2 | T3 | T4 |
| --- | --- | --- | --- | --- |
| Network density | 0.068 | 0.094 | 0.12 | 0.105 |
| Clustering coefficient | 0.234 | 0.257 | 0.295 | 0.272 |
| Average path length | 2.254 | 2.41 | 2.173 | 2.374 |
| Network diameter | 6 | 7 | 6 | 9 |
| Modularity | 0.333 | 0.297 | 0.251 | 0.269 |

Note: T1, no fertilization (0%); T2, low fertilization (25%); T3, half fertilization (50%); T4, full fertilization (100%)
