# Supplementary figures and images for: Effects of chemical fertilization on bacterial community in rhizosphere soil of sugarcane
Source: PLoS One. 2025 Jul 11;20(7):e0327545. doi: 10.1371/journal.pone.0327545 (PMC12250518; doi:10.1371/journal.pone.0327545)

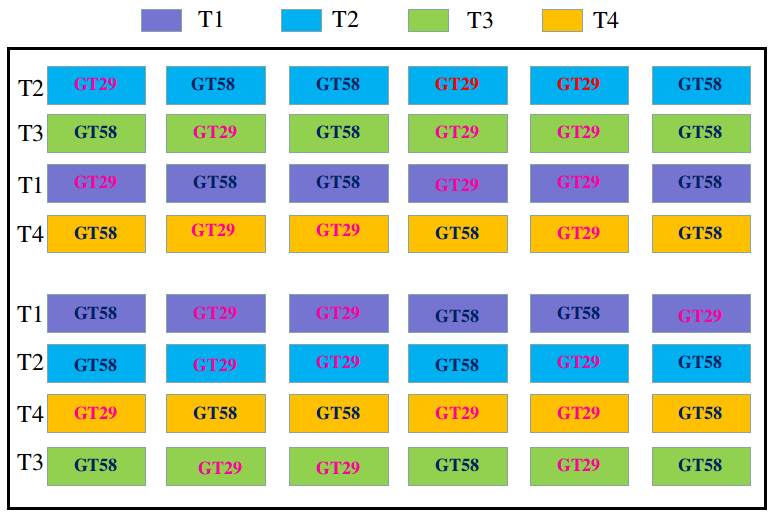


**Supplementary Fig S1. Field experiment design of different fertilization levels**

Supplement: Fig S1 — (DOCX) [file pone.0327545.s004.docx]
